# Integrated analysis of promoter mutation, methylation and expression of *AKT1* gene in Chinese breast cancer patients

**DOI:** 10.1371/journal.pone.0174022

**Published:** 2017-03-16

**Authors:** Jianfu Heng, Xinwu Guo, Wenhan Wu, Yue Wang, Guoli Li, Ming Chen, Limin Peng, Shouman Wang, Lizhong Dai, Lili Tang, Jun Wang

**Affiliations:** 1 The State Key Laboratory of Medical Genetics & School of Life Sciences, Central South University, Changsha, Hunan, China; 2 Sanway Gene Technology Inc., Changsha, Hunan, China; 3 Department of Breast Surgery, Xiangya Hospital, Central South University, Changsha, Hunan, China; 4 Research Center for Technologies in Nucleic Acid-Based Diagnostics, Changsha, Hunan, China; 5 Research Center for Technologies in Nucleic Acid-Based Diagnostics and Therapeutics, Changsha, Hunan, China; University of South Alabama Mitchell Cancer Institute, UNITED STATES

## Abstract

**Background:**

As downstream mediators of *PI3K* /*PTEN* /*AKT* /*mTORC1* pathway, the *AKT* isoforms play critical roles in tumorgenesis. Although the pleiotropic effects of *AKT1* in breast cancer have been reported, the genetic and epigenetic characteristics of *AKT1* promoter region in breast cancer remains to be identified. In this study we aimed to investigate the promoter mutation spectrum, methylation and gene expression pattern of *AKT1* and their relationship with breast cancer.

**Methods:**

By using PCR target sequence enrichment and next-generation sequencing technology, we sequenced *AKT1* promoter region in pairs of breast tumor and normal tissues from 95 unselected Chinese breast cancer patients. The methylation of the promoter region and the expression profile of *AKT1* in the same cohort were detected with bisulfite next-generation sequencing and qPCR, respectively.

**Results:**

We identified 28 somatic mutations in 23 of the 95 (24.2%) breast cancer samples. And 19 of the 28 mutations were located in transcription factor (TF) binding sites. In the 23 patients with somatic mutations, no significant change of methylation or expression was found comparing with other patients. *AKT1* promoter region was significantly hypo-methylated in tumor compared with matched normal tissue (*P* = 0.0014) in the 95 patients. The expression of *AKT1* was significantly suppressed in tumor tissue (*P* = 0.0375). In clinicopathological factor analysis, *AKT1* showed significant hypo-methylation (*P* = 0.0249) and suppressed expression (*P* = 0.0375) in HER2 negative subtype. And a trend of decrease in expression level (*P* = 0.0624) of *AKT1* in the ER negative subtype was observed, which is significantly decreased in basal-like breast tumor (*P* = 0.0328).

**Conclusions:**

Hypo-methylation and suppressed expression of *AKT1* was observed to be associated with breast cancer in our cohort. The methylation and expression of *AKT1* were both significantly associated with HER2 status. The promoter mutation of *AKT1* did not show significant association with its methylation and expression status. These results suggested that the promoter mutation, methylation and gene expression of *AKT1* may play distinct roles in tumorgenesis of breast cancer and the integrated analysis of methylation and expression of *AKT1* might serve as potential biomarkers for diagnosis and classification of breast cancer.

## Introduction

Breast cancer is a leading cause of cancer-related death in women worldwide. According to GLOBOCAN 2012, there are estimated 1.67 million new breast cancer cases diagnosed with over half million deaths each year [1]. In recent years, breast cancer has become the most frequently diagnosed cancer in Chinese women, accounted for 12.2% of global cases and 9.6% of related deaths from breast cancer worldwide [2]. Breast cancer is a heterogeneous disease with distinct histopathological and molecular characteristics. According to the expression pattern of estrogen receptor (ER), progesterone receptor (PR), and human epidermal growth factor receptor-2 (HER2), breast cancer can be classified into four subtypes, including luminal A, luminal B, basal-like and HER2-positive [3]. Environment, genetics and immunological defects are major factors in the etiology of breast cancer [4]. Sporadic breast cancers account for approximately 90–95% of breast cancers, while familiar breast cancers account for the remaining 5–10% due to mutations in genes such as *BRCA1/2* in breast cancer families [5].

Genome-wide studies of DNA sequence, copy number, gene structure and gene expression during the past decade have revealed remarkably diverse aberrations of many genes in breast tumors [6, 7]. *AKT1* is a serine-threonine kinase gene, and involved in many processes including metabolism, proliferation, cell survival, growth and angiogenesis [8–10]. *AKT1* is a downstream mediator of the *PI3K* / *PTEN* / *AKT* / *mTORC1* pathway, which has been suggested to play crucial roles in the development of breast cancer [11, 12]. It has been reported that activation of *AKT1* contributes to resistance to anti-proliferative signals and breast cancer progression [13, 14]. *AKT1* mutations have been reported in 1.4% to 8% (average ~4%) invasive ductal breast carcinoma [15–18], and the oncogenic mutant loci E17K in *AKT1* has been considered as a potential diagnosis biomarker of breast cancer [19].

The mutation and aberrant methylation in the promoter region of tumor suppressor genes, oncogenes, transcription factors and drug response genes could influence the gene expression and play important role in the tumorgenesis, tumor progression and response to treatment [20–22]. However, the *AKT1* mutation and the methylation profile in its promoter region and their role in breast cancer are still not clear. In the present study, we analyzed the *AKT1* promoter mutations with next generation sequencing in breast tumor and matched normal tissues from 95 unselected Chinese breast cancer patients. We also explored the methylation and expression alternation in this cohort with next-generation bisulfite sequencing and qPCR, respectively.

## Material and methods

### Patients and samples

Fresh breast tumor and matched adjacent normal tissues (located at least 2 cm away from the site of tumor tissue) from 95 unselected breast cancer patients were obtained from Xiangya Hospital, Central South University from year 2013 to 2015. The clinicopathological characteristics of patients were shown in Table 1. All breast specimens were reviewed by experienced pathologists. The breast cancer molecular subtypes were characterized based on the guideline of St. Gallen International Expert Consensus [3]. The study was approved by the Ethics Committee at Central South University, Changsha, China. All participants provided written informed consent.

**Table 1:** Clinicopathological characteristics of 95 breast cancer patients.

Characteristics		Number of patients, n (%)	Number of patients with *AKT1* promoter mutations, n (%)^1^	*P* value^2^
Molecular subtype	Basal-like	11 (11.58)	3 (13.04)	0.656
	HER2-enriched	10 (10.53)	2 (8.70)	
	Luminal A	24 (25.26)	5 (21.74)	
	Luminal B	45 (47.37)	13 (56.52)	
	Unknown	5 (5.26)	0 (0)	
ER status	Positive (+)	70 (73.68)	18 (78.26)	0.764
	Negative (-)	25 (26.32)	5 (21.74)	
PR status	Positive (+)	59 (62.11)	14 (60.87)	1
	Negative (-)	36 (37.89)	9 (39.13)	
HER2 status	Positive (+)	23 (24.21)	5 (21.74)	0.951
	Negative (-)	60 (63.16)	15 (65.22)	
	unknown	12 (12.63)	3 (13.04)	
Lymph metastasis	Yes	34 (35.79)	8 (34.78)	1
	No	61 (64.21)	15 (65.22)	
Age	≥50	50 (52.63)	11 (47.83)	0.772
	<50	45 (47.37)	12 (52.17)	

Note:

^**1**^ the percentage was calculated in 23 patients with *AKT1* promoter mutations;

^**2**^ *P* values were calculated between mutated and non-mutated patients using the Chi-square test.

### Primer design

The 5’ promoter sequence for *AKT1* was obtained from UCSC genome browser (http://genome.ucsc.edu/cgi-bin/hgGateway). For the mutation screening, we designed 4 pairs of primers covering the 5’ promoter region sequence of *AKT1* up to 1000 bp using the online software Primer 3 (http://sourceforge.net/projects/primer3). For methylation analysis, the target-specific bisulfite sequencing primers (BSPs) were designed using the online design tool, Methprimer (http://www.urogene.org/methprimer/), with default parameters. The universal sequencing tags were added to the 5’-end of the forward and reverse primers by following the User Guide of Access Array^™^ System for Illumina Sequencing Systems (Fluidigm, South San Francisco, CA, USA). For expression analysis, the cDNA sequence was obtained from the Consensus CDS (http://www.ncbi.nlm.nih.gov/CCDS/CcdsBrowse/). Primers of *GAPDH* and *AKT1* were designed cross exons using Primer 3 and the amplification efficiency was tested as approximate 100%. All the primers (S1 Table) were validated by conventional PCR and PCR products were confirmed for expected size on agarose gels.

### Nucleic acid extraction

The genomic DNA was extracted from the paired tissues using the TIANamp Genomic DNA Kit (TianGen Biotech, Beijing, China) according to the manufacturer’s instruction. Total RNA extraction from the tissue samples was performed with TRIZOL-A reagent (TianGen Biotech, Beijing, China) according to the manufacturer’s instruction. The quality and quantity of all DNA and RNA samples were assessed on Nanodrop 2000 spectrophotometer (Thermo Scientific, Wilmington, DE, USA).

### DNA bisulfite conversion and RNA reverse transcription

Sodium bisulfite conversion of 500 ng genomic DNA was carried out using the EZ DNA Methylation-Lightning^™^ Kit (Zymo Research, Irvine, CA, USA) according to the manufacturer’s instruction. All DNA samples after bisulfite conversion were quantified using a Nanodrop 2000 spectrophotometer (Thermo Scientific, Wilmington, DE, USA). For cDNA synthesis, 500 ng total RNA was reverse transcribed using a Revert Aid 1st Strand cDNA Synthesis Kit (Thermo Scientific, CA, USA) according to the manufacturer’s instruction.

### Target sequence enrichment PCR and next generation sequencing

PCR was used for target enrichment to prepare sequencing libraries. PCR was performed in a Thermal Cycler (Bio-Rad T-100) with 10 μL reaction volume. PCR mix consisted of 1 μL (40 ng/μL) DNA or bisulfite converted DNA sample, 5.9 μL nuclease-free water, 1.8 μL Faststar High Fidelity reaction buffer (Roche, IN, USA), 0.2 μL dNTP, 0.1 μL (10 U/μL) DNA ploymerase (Roche, IN, USA) and 1 μL of each mutation sequencing primers or BSPs (2 μM). The PCR cycling conditions were: 1 cycle of 95°C for 10 min, 40 cycles of 95°C for 15 s, 60°C for 30 s, 72°C for 1 min.

After PCR amplification, sample-specific 10-base barcodes and sequencing tags were added to each PCR product pool according to the User Guide of Access Array^™^ System for Illumina Sequencing Systems (Fluidigm, South San Francisco, CA, USA). Equal volume of each barcoded products were pooled into amplicon libraries and purified using Agencourt AMPure XP system (Beckman Coulter, CA, USA). The product size distribution was examined using Caliper LabChip GX (PerkinElmer, MA, USA). The purified libraries were quantified with Qubit^®^ dsDNA HS Assay Kit (Life Technologies, CA, USA) and sequenced on a MiSeq sequencer using MiSeq Reagent Kit v2, 500 cycles (Illumina, CA, USA).

### Sequencing data alignment for mutation detection and methylation analysis

After sequencing, the paired-end read data were demultiplexed by MiSeq Reporter (v.1.8.1) [23] according to sample specific barcodes with default parameters. After removing low quality reads, the sequences were aligned using BWA (v.0.7.10) with default parameters [24] to the UCSC human reference genome hg19. Germline mutations were called by the GATK Unified Genotyper with paired tumor and normal tissues [25], and somatic mutations were called by MuTect (v.1.1.4) [26]. We used the FIMO tool [27] to scan the promoter region of *AKT1* for significant transcription factor binding sites (TFBSs) occurrences with a *P* value < le-4. The motif position weight matrices (PWMs) for TFBSs from HOCOMOCO (http://autosome.ru/HOCOMOCO/) were used as TFBSs motif input for FIMO. The methylation status and methylation level of each analyzed CpG-site were analyzed and returned from the BiQ Analyzer 3.0 software [28]. The methylation level of *AKT1* was assigned by averaging the methylation level of all CpG sites located in the amplicon for each sample.

### Gene expression analysis

To examine the gene expression of *AKT1*, real-time fluorescence quantitative polymerase chain reaction (qPCR) was performed in a CFX96^™^ Real-Time PCR Detection System (Bio-Rad, CA, USA). The gene expression primers for the *AKT1* and the reference gene *GAPDH* were used for qPCR (S1 Table). All samples were assayed in triplicates. The qPCR mixture consisted of 2 μL of cDNA sample, 2 μL nuclease-free water, 5 μL 2 × SYBR Green PCR master mix (Roche, IN, USA), and 1 μL of each gene specific primer (2 μM). The PCR cycling conditions were: 1 cycle of 95°C for 10 min, 40 cycles of 95°C for 5 s, 60°C for 30 s, and 72°C for 30 s, followed by dissociation curve analysis (65–95°C: increment 0.5°C for 5 s) to verify the amplification of a single product. The threshold cycle (Ct) value was determined using the default setting on the CFX Real-Time PCR Detection System. A mean of the Ct values for *AKT1* and *GAPDH* were calculated for each sample, and expression level of *AKT1* for each sample were determined using the delta Ct (dCt) method as follows: Mean Ct (*AKT1*)—Mean Ct (*GAPDH*), while a higher dCt value suggested lower expression level.

### Statistical analysis

The paired Wilcoxon signed rank test was used to determine the difference of methylation and expression between paired tumor and normal tissues. The Spearman’s rank correlation coefficient test was applied to analyze correlation between methylation and expression for *AKT1* in tumor and normal tissues. The Kruskal-Wallis rank sum test was used to examine the association of methylation or expression level with breast cancer subtypes. The Wilcoxon signed rank test was used to analyze the association of methylation or expression with other clinicopathological factors. Chi-square test was used for categorical data in mutation spectrum analysis. All statistical analysis was performed using R version 3.1.0 (http://www.cran.r-project.org). For all the above analysis, *P* value less than 0.05 was considered as statistically significant.

## Results

### *AKT1* promoter mutation analysis

In the mutation analysis, we sequenced 1000 bp promoter region of *AKT1* from 95 pairs of Chinese breast cancer tissues. We obtained high quality sequencing data with average gene read depth of 500 reads per sample. After applying the threshold at mutant allele fraction (AF) of >5%, totally 28 somatic mutant loci were detected in 23 of the 95 (24.2%) breast cancer patients, and most of the mutations were rare mutations. We predicted the TF binding site (TFBS) using the software FIMO. It revealed that in the 28 somatic mutations, 19 loci were located in TFBSs and 16 variants were predicted to result in loss or gain of TFBSs (Table 2). No germline mutation in the *AKT1* promoter region was discovered in this cohort.

**Table 2:** The *AKT1* methylation, expression and TFBS status of patients with promoter somatic mutations.

Mutation Position (hg19)	Sample ID	Subtype	Mutant allele fraction (%)	Expression (dCt)		Methylation (%)		RefSeq_TF	Loss of TFBS	Gain of TFBS
				Tumor	Normal	Tumor	Normal			
chr14:g. 105262255G/A	S32_4	Luminal A	57	4.07	2.93	7.97	6.89	.	.	
chr14:g. 105262441G/A	S8_3	Luminal B	16.87	-0.85	3.59	9.32	11.76	*EGR1*; *KLF5*; *NR2C2*; *RREB1*; *SP1*; *SP2*	*RREB1*	.
chr14:g. 105262373C/T	S1_4	Luminal B	13.6	4.05	4.27	6.82	7.21	.	.	
chr14:g. 105262491G/A	S16_2	Luminal B	12.75	4.81	4.00	8.64	7.70	*EGR1*; *PAX5*; *PLAG1*	*PLAG1*	*ZNF263*
chr14:g. 105262266T/C	S14_3	Luminal B	12.62	4.60	4.10	5.02	7.15	.	.	.
chr14:g. 105262526A/G	S2_4	HER2	12.44	3.00	3.66	7.93	8.18	.	.	*HNF4A*
chr14:g. 105262534T/G	S23_2	Luminal B	11.79	4.93	5.52	7.09	6.45	.		*PLAG1*; *NR1H2*; *RXRA*
chr14:g. 105262639C/T	S13_3	Luminal B	9.66	2.85	4.12	4.04	7.45	*REST*; *THAP1*	*REST*; *THAP1*	.
chr14:g. 105262522C/T	S2_4	HER2	8.52	3.00	3.66	7.93	8.18	.	.	.
chr14:g. 105262269C/T	S10_3	Luminal B	8.32	2.11	3.91	8.14	7.00	.	.	*HOXA5*; *TLX1*; *NFIC*
chr14:g. 105262286C/T	S10_3	Luminal B	8.09	2.11	3.91	8.14	7.00	.	.	.
chr14:g. 105262333T/C	S23_2	Luminal B	7.79	4.93	5.52	7.09	6.45	.	.	.
chr14:g. 105262931C/T	S6_4	Luminal B	7.59	3.89	3.52	9.38	8.95	*SMAD2*; *SMAD3*; *SMAD4*	*SMAD2*; *SMAD3*; *SMAD4*	.
chr14:g. 105262991C/T	S8_2	Luminal A	7.5	3.23	3.19	6.15	8.65	*PLAG1*	*PLAG1*	.
chr14:g. 105262531T/C	S42_4	Luminal A	6.86	2.55	4.00	6.59	11.18	.	.	.
chr14:g. 105262293G/A	S34_4	Luminal B	6.69	4.46	3.34	4.97	8.14	.	.	*RREB1*
chr14:g. 105262937C/T	S38_4	Basal-like	6.45	2.06	3.88	9.80	8.06	*INSM1*	*INSM1*	*EWSR1*-*FLI1*
chr14:g. 105262848A/G	S2_4	HER2	6.30	3.00	3.66	7.93	8.18	*CTCF*	.	.
chr14:g. 105262438C/A	S45_4	Luminal A	6.29	4.53	3.41	9.53	6.43	*EGR1*; *KLF5*; *NR2C2*; *RREB1*; *SP1*; *SP2*	*NR2C2*	*E2F4*; *MZF1_5–13*
chr14:g. 105262295C/T	S21_4	Luminal B	6.28	4.40	4.56	7.50	6.65	.	.	.
chr14:g. 105262829C/A	S10_2	HER2	6.25	4.32	5.14	8.63	5.68	*HNF4A*; *HNF4G*	*HNF4G*	.
chr14:g. 105262338A/G	S26_4	Luminal B	6.18	4.38	3.69	6.72	8.34	.	.	.
chr14:g. 105262863C/A	S2_4	HER2	6.09	3.00	3.66	7.93	8.18	*BATF*; *JUN*; *JUN* (var.2)	*JUN* (var.2)	
chr14:g. 105262419T/C	S35_4	Luminal B	5.64	3.22	4.83	10.60	7.85	*EGR1*	.	*SP2*
chr14:g. 105262542G/A	S37_4	Basal-like	5.52	2.98	4.31	11.37	7.17	*EBF1*; *ZNF263*	*EBF1*	.
chr14:g. 105262975C/T	S31_4	Basal-like	5.47	6.19	4.68	9.75	8.62	*EGR1*; *KLF5*; *SP1*; *SP2*	*SP1*; *KLF5*; *SP2*; *EGR1*	.
chr14:g. 105262503G/A	S27_4	Luminal A	5.46	3.67	3.91	5.35	10.23	*PAX5*	.	.
chr14:g. 105262690G/A	S17_3	Luminal B	5.42	5.78	3.66	7.64	10.35	*ZNF263*	.	.

The clinicopathological characteristics of 23 patients with *AKT1* promoter mutation were shown in Table 1. Chi-square test was used to detect if mutation in promoter region is associated with clinical characteristics. No significant association was found between the mutations and these clinicopathological factors.

### *AKT1* promoter methylation and gene expression analysis

**Fig 1:**
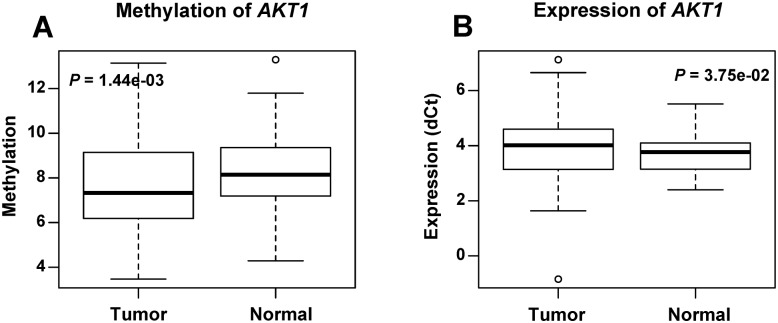
The methylation and expression level of *AKT1* in tumor and normal tissues from 95 breast cancer patients. Boxplots show the average methylation (A) and expression (B) of *AKT1* in breast tumor and normal tissues, and *P* values were calculated using the paired Wilcoxon signed rank test.

Sequencing of bisulfite-converted genomic DNAs revealed that the *AKT1* promoter region were hypo-methylated in breast tumor tissues compared with the matched adjacent normal tissues (Fig 1). Average methylation level of the 10 CpG sites within 172 bp of *AKT1* promoter region showed significant difference between the 95 tumor (7.49%) and matched normal tissues (8.35%) (*P* = 0.00144). The *AKT1* expression analysis showed that average gene expression level (dCt) of *AKT1* in tumor is lower than normal tissues. A significant difference (*P* = 0.0375) between the tumor and normal tissues was observed (Fig 1). These results showed significant lower methylation level and significant lower gene expression level (high dCt) in tumor tissues than normal tissues, but no significant *cis* correlation was found between the methylation and the expression level (*P* = 0.160, R^2^ = 0.010) using Spearman’s rank correlation analysis (S1 Fig).

### Correlation of the methylation and expression level of *AKT1* gene with patient clinicopathological characteristics

**Fig 2:**
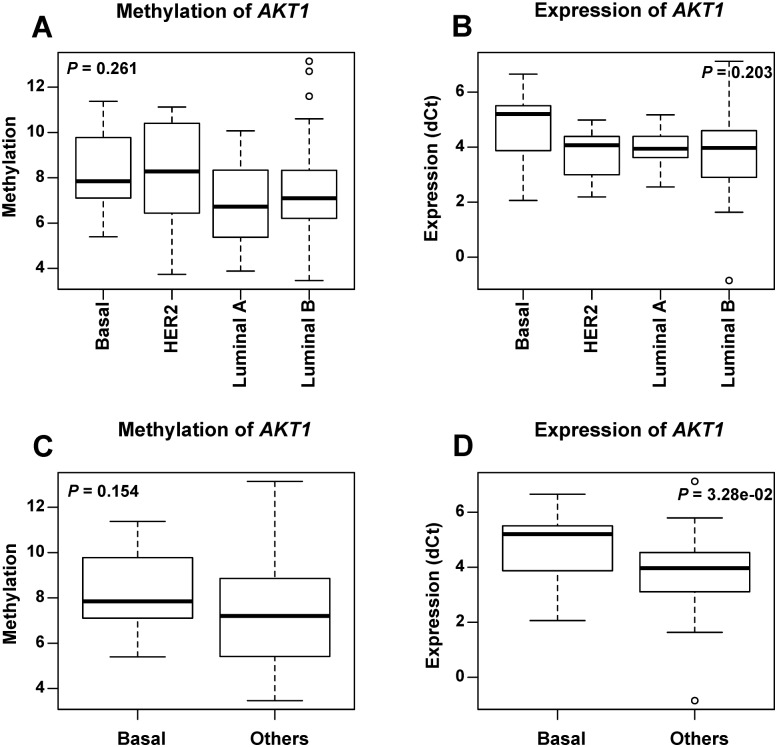
The methylation and expression level of *AKT1* in breast cancer subtypes. Boxplots show the average methylation (A) and expression (B) level of *AKT1* in four different breast cancer subtypes, as well as methylation (C) and expression (D) in basal-like and non-basal-like breast tumor tissues. *P* values were calculated using the Kruskal—Wallis rank sum test for breast cancer subtypes and the Wilcoxon signed rank test for basal-like and non-basal-like breast tumor tissues.

**Fig 3:**
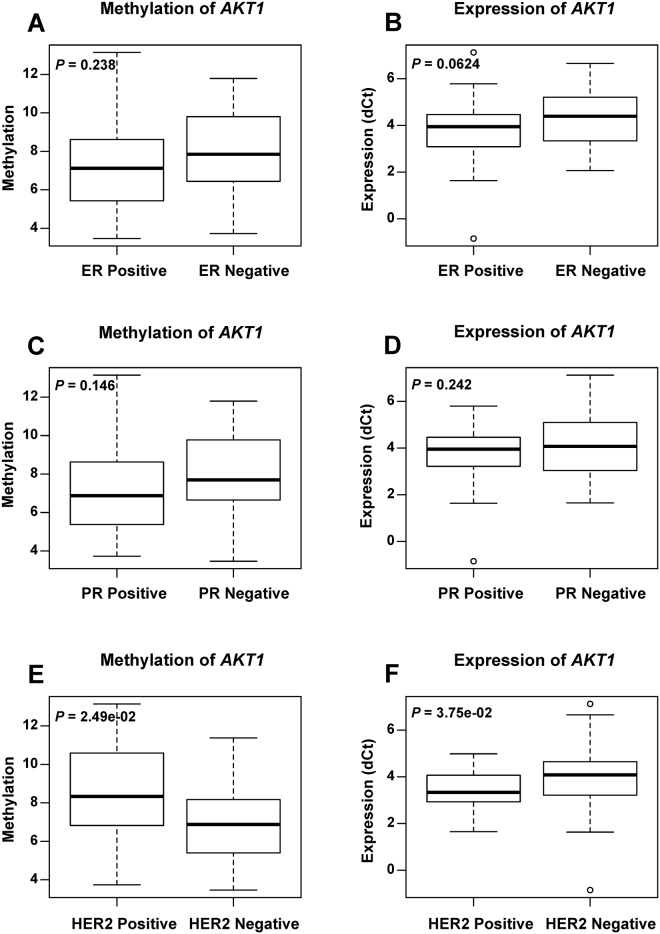
The methylation and expression level of *AKT1* in breast cancer patients according to ER/PR/HER2 status. Boxplots show the average methylation (A, C, E) and expression (B, D, F) level of *AKT1* between ER-positive and ER-negative subgroups, between PR-positive and PR-negative subgroups, as well as between HER2-positive and HER2-negative subgroups. *P* values were calculated using the Wilcoxon signed rank test.

We analyzed the *AKT1* methylation and expression level according to the clinicopathological characteristics of the breast cancer patients (S2 Table). No significant difference either in the methylation or in gene expression was observed among four breast cancer subtypes. However, it was obvious that the basal-like breast cancer subgroup showed the lowest mean expression level of *AKT1* (Fig 2). In the comparison of *AKT1* methylation and expression between basal-like tumor and other subtypes, it revealed significant lower *AKT1* expression (*P* = 0.0328) in basal-like breast cancer than other subtypes (Fig 2). The analysis of the methylation and expression according to ER, PR and HER2 (Fig 3) status showed that the methylation (*P* = 0.0249) and expression (*P* = 0.0375) of *AKT1* were significantly associated with HER2 status. The hypo-methylation and suppressed expression of *AKT1* were associated with HER2 negative tumors. The expression level in ER negative breast cancer was lower than that in the ER positive subtypes, although it was not significantly different (*P* = 0.0624). The low expression level of *AKT1* in ER negative and HER2 negative tumor is consistent with low expression in basal-like tumor which is ER and HER2 negative. In addition, no significant difference was observed according to the status of age and lymph metastasis (data not shown).

### *AKT1* methylation and expression analysis in patients with promoter mutations

**Fig 4:**
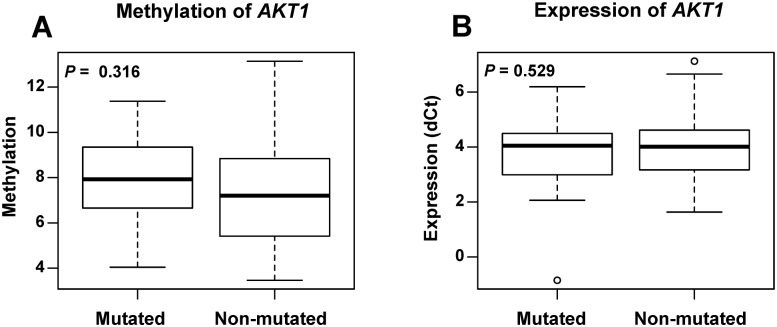
The methylation and expression level of *AKT1* in breast cancer patients with *AKT1* mutations. Boxplots show the average methylation (A) and expression (B) level of *AKT1* in patient tumor tissues with or without *AKT1* promoter mutations. *P* values were calculated using the Wilcoxon signed rank test.

Totally, 23 tumor tissue samples carried 28 somatic mutations (Table 2) in the promoter region of *AKT1*. We compared the methylation level between mutated and non-mutated tumor samples. No significant difference of methylation was found between these two groups of tumors (*P* = 0.316) (Fig 4). In addition, we looked at the expression level of *AKT1* in the samples with mutations. The expression was depressed in 9 tumors, and elevated in other 14 tumors (Table 2). However, no significant expression difference was found between mutated and non-mutated tumors (*P* = 0.529) (Fig 4). We did not observe any significant association of *AKT1* promoter mutation with its methylation and expression.

## Discussion

Aberrations of phosphatidylinositol 3-kinase PI3K/AKT pathway were frequently observed in cancer. Mutations and aberrant methylation of members in PI3K/AKT pathway play critical roles to regulate gene expression involved in tumorgenesis and metastasis [8]. This pathway therefore emerges as one of the promising targets of anticancer drugs in the near future. In this study, we investigated the *AKT1* promoter mutation spectrum, methylation pattern and their potential role on its expression. Hypo-methylation and decreased expression of *AKT1* were observed significantly associated with breast cancer in this study.

Since the E17K mutation of *AKT1* was firstly identified as a potential biomarker in breast cancer, the coding region of *AKT1* has become the hotspot of mutation detection [18]. However, the mutation spectrum in promoter region of *AKT1* in breast cancer is still unclear. In the mutation analysis, we found somatic mutation (AF>5%) in 24.2% of breast cancer patients (23 in 95) indicating that *AKT1* promoter mutation may be a frequent event in breast cancer. And mutation loci distributed in all subtypes of breast cancer. These results were different from the previous meta analysis on *AKT1* mutation, in which only 3.8% *AKT1* mutation was detected in breast cancer patient and restricted to hormone receptor—positive cancers [18, 29, 30]. It could be explained by the following two aspects. Firstly, the breast cancer gene mutation pattern may be different in races. Secondly, the reported sequence target was the exon regions, while our target was the promoter region of *AKT1*. It implied that the promoter region may be the mutation hotspot of *AKT1* in Chinese breast cancer patients. Wang et al [31] reported three SNPs (rs2494750 G>C, rs2494752 A>G, and rs10138227 C>T) in *AKT1* promoter region in gastric adenocarcinoma. *AKT1* rs2494750 G>C located in our target sequence, which we also observed in this study (data not shown). In addition, we analyzed the TFBS change and the corresponding expression alternation in the 23 tumors harbored somatic mutations. *AKT1* expression was elevated in 14 and reduced in 9 breast tumors. In general, we did not find *AKT1* promoter mutations significantly associated with its gene expression in the 95 breast cancer patients. These observations still need further validation in large cohorts.

Increasing evidence indicated that tumorgenesis depends on not only the acquisition of genetic alterations, but also epigenetic perturbations, which adds an important layer of transcriptional control to the cancer genome. It has been shown that DNA methylation at gene promoter regions plays a critical role in maintaining silencing of tumor suppressor genes in tumors, including breast cancer. The promoter hyper/hypo-methylation is linked and perhaps directly contributes to tumorgenesis, invasion, metastasis, and chemotherapeutic resistance [32]. Mutation and increased phosphorylation of *AKT1* were identified in different types of cancers, including melanoma, breast, esophageal, colorectal, endometrial, ovarian, and non-small cell lung cancers [33]. In the present study, *AKT1* was significantly hypo-methylated and less expressed in the breast tumor compared with the matched normal tissue, but no significant *cis* correlation was found between methylation and expression. Similar situation was observed in several studies, which supported the notion that methylation is sufficient but not necessary for their inactivation of gene expression [34, 35]. We analyzed and checked the methylation and expression data of *AKT1* in TCGA from MethHC database (http://methhc.mbc.nctu.edu.tw/php/index.php). *AKT1* also showed significant hypo-methylation in breast tumor tissues in TCGA, which is consistent with our results. *AKT1* showed significant difference of expression between tumor and normal tissue in TCGA but with higher expression in tumor. The discrepancy observed here is most likely related to the differences in detection methods, stage or type of breast tumor, and even the differences in race or ethnicity [36].

In the subtype analysis, we found that *AKT1* expression was suppressed in basal-like tumors and HER2 negative tumors, comparing with that in Lumina A and Lumina B subtypes. Previous publication has demonstrated that constitutively activated AKT1 expression inhibited the basal-like breast cancer cell line MDA-MB231 cells proliferation [37]. In addition, over-expressed pAKT1 in HER2 positive breast cancer was associated with poor prognosis [38, 39], indicating a post-translational modification mechanism for AKT1 in breast cancer. Our observation suggested that *AKT1* expression aberrations likely play distinct role in the pathogenesis of different breast cancer subtypes [18]. In the comparison of the methylation level between the mutated and non-mutated specimens, no significant difference was found. This suggested that the *AKT1* promoter mutation was not an influence factor on its methylation and they may play distinct role in tumorigeneis.

In summary, *AKT1* promoter mutation and methylation alternation were observed commonly in our cohort of Chinese breast cancer patients. The promoter hypo-methylation and decreased gene expression were associated with breast tumor. The *AKT1* promoter mutation, methylation and expression may play distinct roles in breast cancer and could be potential biomarkers for breast cancer diagnosis and classification. The decrease of *AKT1* expression was observed in basal-like and HER2 negative breast tumor, which could benefit for diagnosis and targeted therapy of basal-like breast cancer based on subtype. The results of present study were found in a relative small cohort, which may need further validation. Consequent confirmation of our discoveries in a larger breast cancer cohort might lead to a better understanding of breast cancer pathogenesis and benefit breast cancer early detection and classification.

## Supporting information

S1 Fig Scatter plot for methylation and expression of *AKT1*. Spearman’s rank correlation test was used for the *cis* correlation analysis between methylation and expression. Normal tissue in green and tumor in red.
(TIF)

S1 Table Primers for *AKT1* promoter mutation, expression and methylation analysis. (DOCX)

S2 Table The raw data including sample information, methylation, expression, and *AKT1* promoter mutation status. (XLSX)
