# Supplementary figures and images for: Integrated analysis of promoter mutation, methylation and expression of AKT1 gene in Chinese breast cancer patients
Source: PLoS One. 2017 Mar 16;12(3):e0174022. doi: 10.1371/journal.pone.0174022 (PMC5354459; doi:10.1371/journal.pone.0174022)

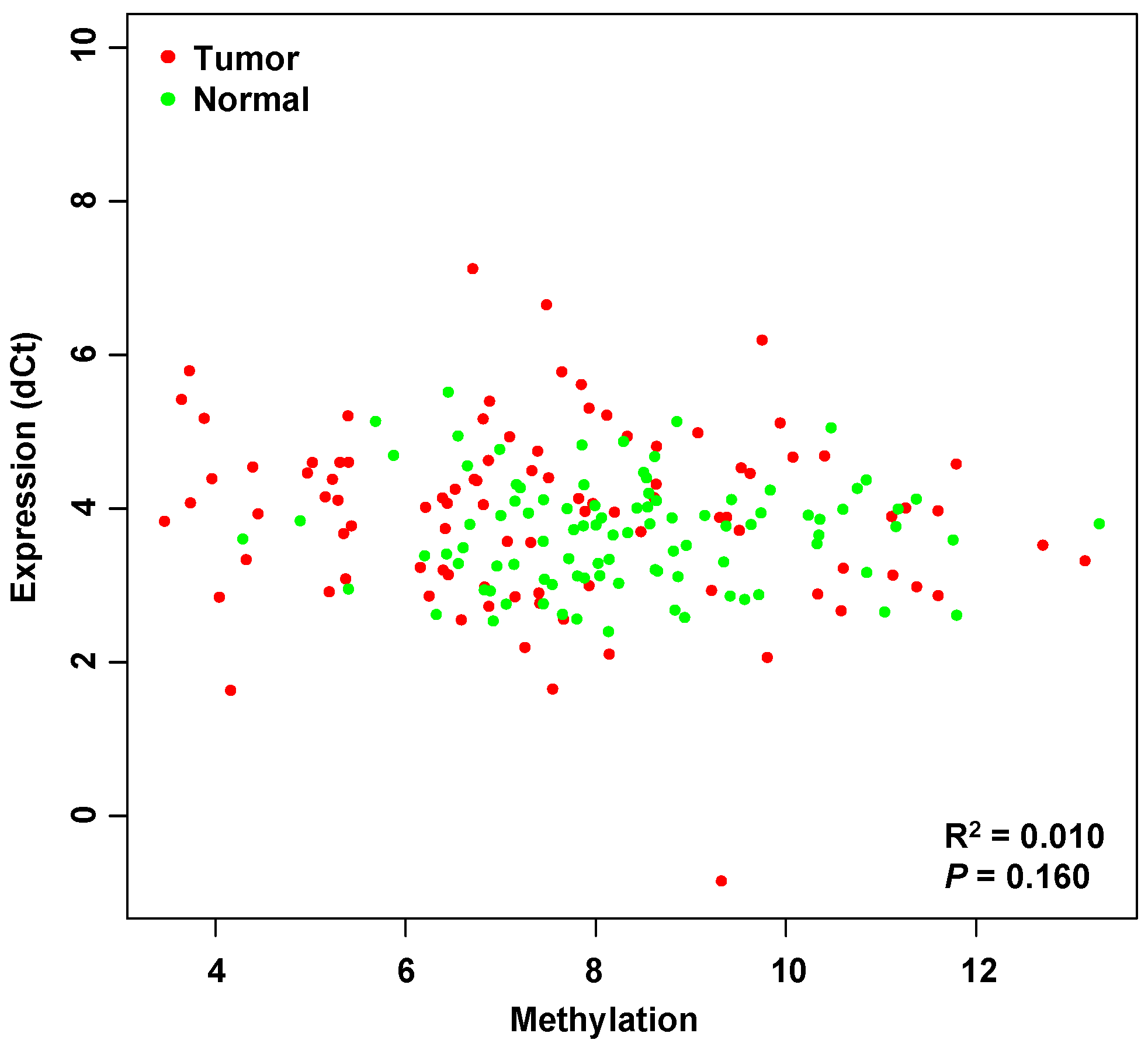

Supplement: S1 Fig — Spearman’s rank correlation test was used for the cis correlation analysis between methylation and expression. Normal tissue in green and tumor in red. (TIF) [file pone.0174022.s001.TIF]
