# Supplementary material for: Integrated analysis of promoter mutation, methylation and expression of AKT1 gene in Chinese breast cancer patients
Source: PLoS One. 2017 Mar 16;12(3):e0174022. doi: 10.1371/journal.pone.0174022 (PMC5354459; doi:10.1371/journal.pone.0174022)
Supplement: S1 Table — (DOCX) [file pone.0174022.s002.docx]

**S1 Table. Primers for *AKT1* promoter mutation, expression and methylation analysis**

| **Primer name** | **Sequence** | **Amplicon Length** | **Application** |
| --- | --- | --- | --- |
| *AKT1*_P_1F | TGAACTCACTCTGGCTGAAAA | 320 bp | Promoter mutation sequencing |
| *AKT1*_P_1R | CAGAGCCCTCCCTGCTGG |  |  |
| *AKT1*_P_2F | CAGTGGACTTCGGACTGGG | 317 bp |  |
| *AKT1*_P_2R | AAGACCTTGTGCCTCTGGG |  |  |
| *AKT1*_P_3F | CTCCAACTGACCTCCTGTCC | 267 bp |  |
| *AKT1*_P_3R | CCTCAGTTTCCCCGTCTGTA |  |  |
| *AKT1*_P_4F | GGATTCGTCCCTGACCTGTC | 182 bp |  |
| *AKT1*_P_4R | CAGCTCCCGACGACCTGG |  |  |
| *AKT1*-F | TTGTGAAGGAGGGTTGGCTG | 159 bp | Expression |
| *AKT1*-R | GCGCCACAGAGAAGTTGTTG |  |  |
| *GAPDH*-F | ATGTTCGTCATGGGTGTGAA | 160 bp | Expression reference |
| *GAPDH*-R | CAGTGATGGCATGGACTGT |  |  |
| *AKT1*-bsp-F | TAGAGGTATAAGGTTTTGGGATTG | 172 bp | Methylation sequencing |
| *AKT1*-bsp-R | TAACCCTAACAAAAAAAACCC |  |  |
